# DEPDC1 is required for cell cycle progression and motility in nasopharyngeal carcinoma

**DOI:** 10.18632/oncotarget.18868

**Published:** 2017-06-29

**Authors:** Xuefei Feng, Chundong Zhang, Ling Zhu, Lian Zhang, Hongxia Li, Longxia He, Yan Mi, Yitao Wang, Jiang Zhu, Youquan Bu

**Affiliations:** ^1^ Department of Otolaryngology, The First Affiliated Hospital of Chongqing Medical University, Chongqing 400016, China; ^2^ Department of Biochemistry and Molecular Biology, Chongqing Medical University, Chongqing 400016, China

**Keywords:** DEPDC1, cell cycle, mitosis, nasopharyngeal carcinoma

## Abstract

DEP domain containing 1 (DEPDC1) is a newly identified cancer-related and cell cycle related gene and has been demonstrated as a novel therapeutic target for bladder cancer. However, the functional involvement and therapeutic potential of DEPDC1 in nasopharyngeal carcinoma (NPC) remains unclear. Our results showed that DEPDC1 was overexpressed at both mRNA and protein levels in NPC tissues compared with normal or non-tumor tissues. The siRNA-mediated DEPDC1 depletion resulted in significant inhibition of proliferation and delay in cell cycle progression in both NPC cell lines, CNE-1 and HNE-1. Detailed analysis with indirect immunofluorescence assays revealed that DEPDC1 depletion caused significant mitotic arrest accompanied with mitotic defects such as multipolar spindles and multiple nuclei followed by apoptotic cell death. Notably, DEPDC1 depletion also reduces migration and invasion ability in both cell lines. Consistent with its regulatory role in NF-κB pathway, knockdown of DEPDC1 caused significant upregulation of A20 and downregulation of mutiple NF-κB downstream target genes implicated in proliferation and tumorigenesis (c-Myc, BCL2, CCND1, CCNB1 and CCNB2), and metastasis (MMP2, MMP9, ICAM1, vimentin, Twist1). Moreover, *in vivo* study demonstrated that DEPDC1 knockdown also caused significant inhibition of tumor growth in the NPC xenograft nude mouse model. Taken together, our present study demonstrated that DEPDC1 is essentially required for the accelerated cell cycle progression and motility in NPC cells, and strongly suggested that DEPDC1 may serve as a novel therapeutic target in NPC.

## INTRODUCTION

Nasopharyngeal carcinoma (NPC) is a type of head and neck cancer and shows unique geographical and ethnic distribution with high incidence in southeast Asia especially in the southern China [1–3]. NPC often invades adjacent regions and metastasizes to regional lymph nodes and distant organs. Distant metastasis, most commonly to lung, is the main cause of treatment failure, and the incidence of distant metastasis is higher in patients with advanced disease [4, 5]. Of note, prognosis could be quite different even in NPC patients with the same clinical stage which are relevant to tumor-specific biological characteristics such as proliferation [6, 7]. Therefore, elucidation of the molecular mechanisms underlying the tumorigenesis, invasion and metastasis of NPC is critical for the treatment of this disease.

DEP domain containing 1 (DEPDC1) is a recently identified novel tumor-related gene and highly conserved from *Caenorhabditis elegans* to human [8–10]. DEPDC1 is the human counterpart of LET-99 in Caenorhabditis elegans. In human, DEPDC1 gene is located at 1p31.3. Northern blot and 5′-RACE analyses revealed that there exist two DEPDC1 transcriptional variants, both of which are overexpressed in bladder cancer cells [9]. Detailed sequence analysis found that the longer variant consists 12 exons encoding the longer isoform of DEPDC1-V1 with 811 amino acid residues whereas the shorter variant lacks an in-frame exon in the central coding region hence resulting in the shorter isoform of DEPDC1-V2 with 527 amino acid residues. The DEP (Dishevelled, EGL-10, Pleckstrin) domain exists in the N-terminal regions of both isoforms [9].

DEPDC1 was firstly reported to be aberrantly overexpressed in bladder cancer and has an important role in the bladder cancer development [9]. Subsequent studies showed that DEPDC1 was also overexpressed in breast cancer, hepatocelluar carcinomas, multiple myeloma, and prostate cancer [11–14]. Consequently, knockdown of DEPDC1 inhibited growth and induced apoptosis in bladder cancer, myeloma, and prostate cancer cells [9, 12, 14, 15]. Of note, targeting DPEDPC1 with a cell-permeable peptide achieved optimistic therapeutic effects in bladder cancer both *in vitro* and *in vivo* [8].

Recently, we have for the first time further identified DPEDC1 to be a novel cell cycle related gene that regulates mitotic progression [15]. Expression profiling with synchronized cells showed that DEPDC1 is highly expressed in the mitotic phase during cell cycle. Knockdown of DEPDC1 caused a significant mitotic arrest with several mitotic defects, suggesting its critical role in proper mitotic progression. Of note, early studies demonstrated that LET-99 acts downstream of the polarity protein PAR-3 to determine mitotic spindle position in early *C. elegans* embryos [10, 16, 17]. Intriguingly, a recent elegant study further showed that DEPDC1/LET-99 is an evolutionarily conserved mediator for anti-tubulin drug-induced apoptosis, and DEPDC1 could be an additional determinant for therapy response upstream of MCL1 [18]. Given the well-known close relationship between cell cycle dysregulation and tumorigenesis, DEPDC1 might be widely involved in the development of many types of cancers.

Currently, the expression and functional involvement of DEPDC1 in NPC remains unclear. The aim of this study is to investigate the expression profile and functional implication of DEPDC1 as well as its therapeutic potential in NPC. We have for the first time found that DEPDC1 is highly expressed in NPC tissues relative to non-tumor tissues, and knockdown of DEPDC1 caused significant growth suppression of NPC cells *in vitro* and *in vivo*, and also inhibited cell migration and invasion in NPC cells.

## RESULTS

### DEPDC1 is overexpressed in NPC tissue

**Figure 1:**
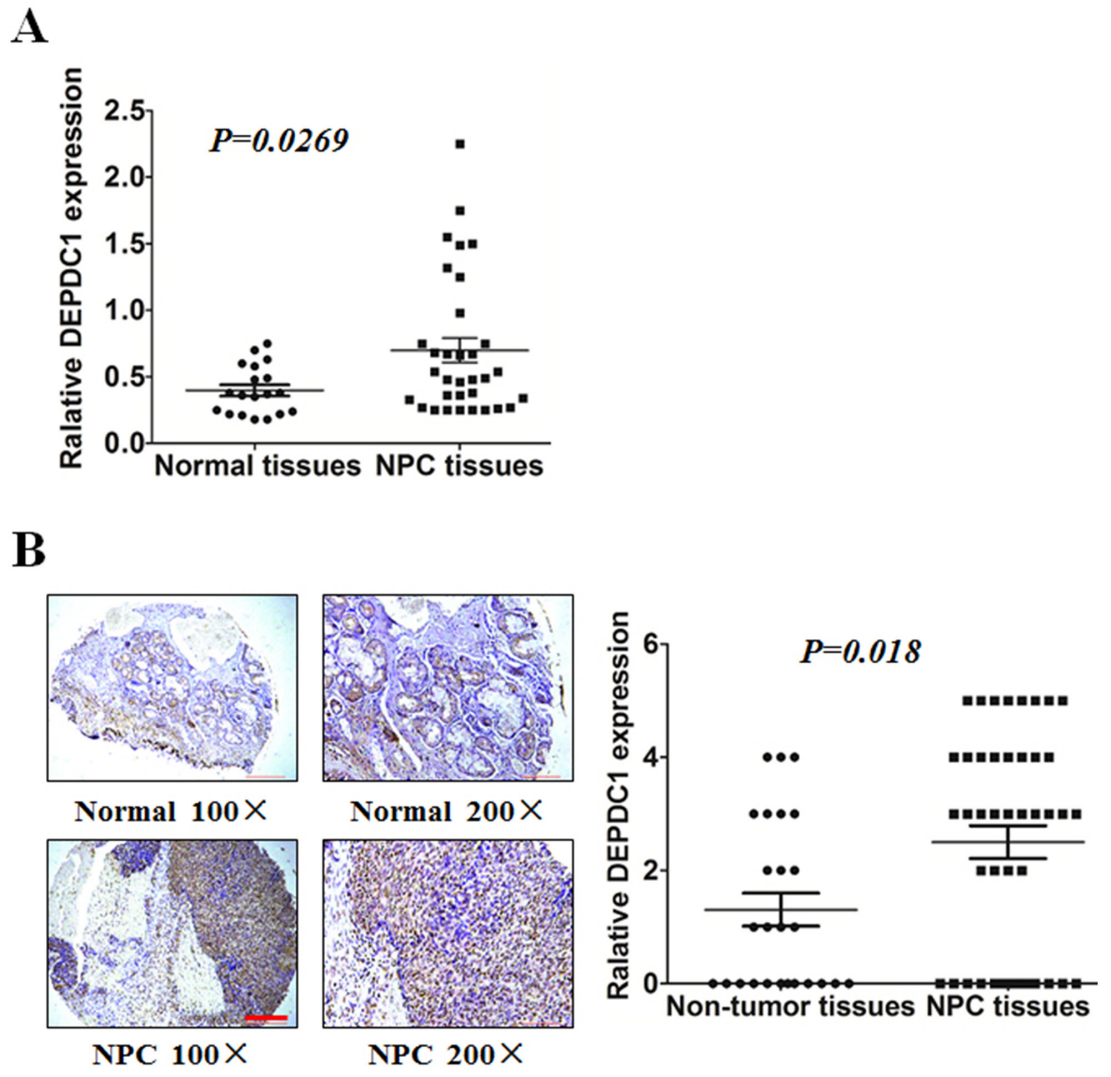
DEPDC1 is overexpressed in NPC **(A)** Expression of DEPDC1 in 18 normal and 33 NPC tissues was analyzed by qRT-PCR using fresh frozen tissues (*p=0.0269*, independent samples t test). **(B)** Representative image of DEPDC1 expression in NPC tissues and non-tumor tissues by immunohistochemistry (100× and 200× magnification) and quantitative immunohistochemistry analysis for DEPDC1 expression in 44 NPC tissues and 26 non-tumor tissues (2 adjacent normal nasal tissues, 12 polyp, 6 hyperplasia, and 6 inflammation). Scale bar 200 μm (100×), 100μm (200×) (*p=0.018*, Mann-Whitney test).

To compare the expression of DEPDC1 in NPC tissues and normal nasopharyngeal tissues, we performed RT-PCR on 51 tissues with biopsy, including 33 patients with nasopharyngeal carcinoma and 18 healthy controls. DEPDC1 was significantly upregulated at mRNA level in NPC samples compared with normal nasopharyngeal tissue (Figure 1A). Furthermore, we evaluated endogenous DEPDC1 expression by immunohistochemistry using a NPC tissue microarray. As shown in Figure 1B, DEPDC1 was predominantly expressed in the nucleus. Compared with the non-tumor tissues, NPC tissuses showed much stronger staining intensity. Strong staining of DEPDC1 protein was detected in 26 (59%) of 44 NPC, but only in 7 (27%) of 26 non-tumor tissues. However, probably due to the limited number of NPC tissue samples, statistical analysis revealed that DEPDC1 was not correlated with clinic pathological parameters such as gender, age, clinical stages and TNM classification (Supplementary Table 3).

### DEPDC1 depletion inhibits cell proliferation in NPC cells

**Figure 2:**
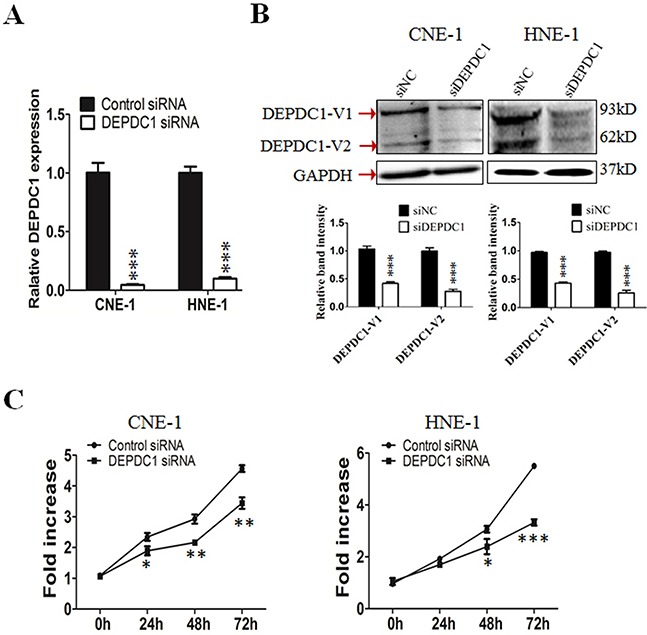
DEPDC1 knockdown inhibits NPC cells proliferation **(A & B)** siRNA-mediated DEPDC1 depletion. CNE-1 and HNE-1 cells were transiently transfected with the control siRNA and DEPDC1 siRNA, respectively. Fourty-eight hours after transfection, total RNA and whole cell lysates were prepared. The expression of DEPDC1 was determined at both mRNA and protein levels by qRT-PCR **(A)** and immunoblotting **(B)**, respectively. Optical densities (O.D.) of individual bands on immunoblots were quantified. Data are expressed as the ratio of the DEPDC1 : GAPDH optical densities in DEPDC1 siRNA group relative to that in NC siRNA group and are representative of three separate experiments (mean ± S.D.). GAPDH was use as an internal control (P*** < *0.001*, Student's t-test). **(C)** DEPDC1 depletion inhibits NPC cell proliferation. Immediately after siRNA transfection, cell proliferation was measured at the indicated time points by CCK-8 assay in CNE-1 and HNE-1 cells, respectively. The y-axis represents the average fold increase in cell numbers. The error bars represent the standard errors of three independent experiments (P* < 0.05 P** < 0.01 P*** < *0.001*, Student's t-test).

To understand the functional significance of DEPDC1 in NPC, we depleted the DEPDC1 expression via siRNA-mediated silencing in two different NPC cell lines, CNE-1 and HNE1. RT-PCR and Western blot analysis confirmed that the DEPDC1 expression was significantly depleted at both mRNA and protein levels in both cell lines (Figure 2A and 2B). We then examined whether silencing of DEPDC1 could affect the proliferation of NPC cells. The proliferation assay results clearly indicated that knockdown of DEPDC1 led to significant inhibition of proliferation in both CNE1 and HNE-1 cells (Figure 2C).

### DEPDC1 depletion delays cell cycle progression in NPC cells

**Figure 3:**
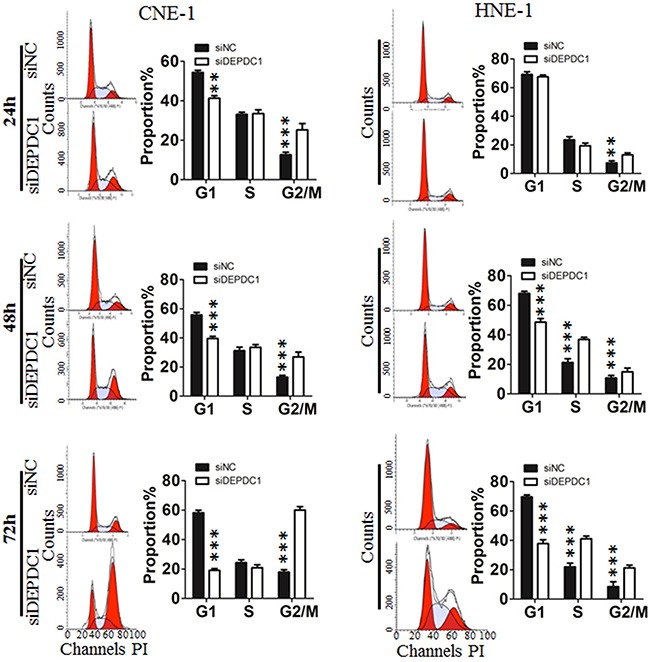
DEPDC1 depletion affects cell cycle progression CNE-1 and HNE-1 Cells were transfected with negative control siRNA or DEPDC1 siRNA, and then subjected to cell cycle distribution analysis by flow cytometry at 24 h, 48h and 72h after transfection, respectively. Cell cycle distribution data were quantified based on at least three independent experiments (P* < 0.05 P*** < *0.001*, using Student's t-test).

To investigate the potential role of DEPDC1 in cell cycle progression, we analyzed the cell cycle distribution at 24 h, 48 h and 72 h after siRNA transfection, respectively. As shown in Figure 3, silencing of DEPDC1 expression resulted into a significant G2/M phase arrest in CNE-1 and a moderate S and G2/M phase arrest in HNE-1 cells. With the extension of transfection time, the effect is much more obvious. Seventy-two hours after siRNA transfection, about 60% of DEPDC1-knocked down HNE-1 cells were arrested in G2/M phase compared with about 20% in control siRNA-transfected cells, and 20% of DEPDC1-knocked down CNE-1 cells compared with 10% in control cells (Figure 3).

**Figure 4:**
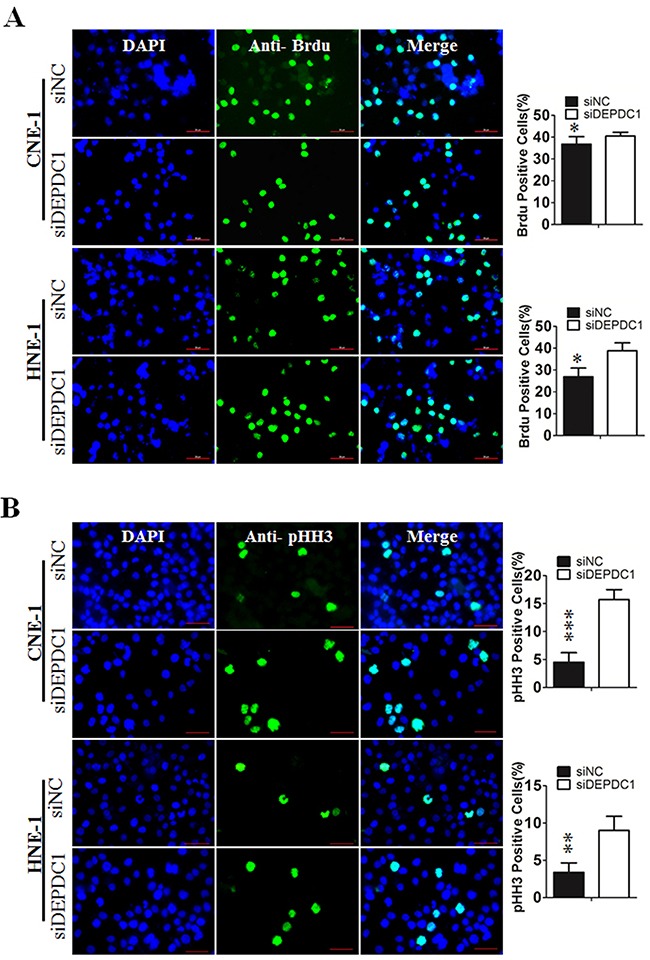
DEPDC1 depletion causes mitotic cell cycle arrest **(A)** CNE-1 and HNE-1 cells were seeded and transfected with negative control siRNA or DEPDC1 siRNA. Forty-eight hours after transfection, DNA biosythensis was labelled by BrdU (green). DAPI was used to stain cell nuclei (blue). BrdU positive cells were counted and subjected to statistical analysis (P* < *0.05*, Student's t-test). Scale bar 50μm. **(B)** CNE-1 and HNE-1 cells were transiently transfected with siRNA as in Figure 4A. Forty-eight hours after transfection, cells were fixed and stained with anti-phospho-histone H3 antibody (green). The pHH3 positive cells were counted and subjected to statistical analysis (P** < *0.01*, Student's t-test).

To determine at which stage of the cell cycle phase DEPDC1-knocked down cells were arrested, NPC cells were incubated with BrdU, and then subjected to immunofluorescence staining with anti-BrdU antibodies. At the same time, we also performed indirect immunofluorescence staining with antibody against phospho-histone H3, a well-established mitotic marker. Consistent with the results of cell cycle analysis, BrdU positive cells in DEPDC1 knockdown group was slightly increased as compared with control group (CNE-1: 36.78%±0.6% in control group VS 40.84%±0.34% in DEPDC1 knockdown group; HNE-1: 28.55%±0.46% in control group VS 36.84%±0.44% in DEPDC1 knockdown group) (Figure 4A). As shown in Figure 4B, knockdown of DEPDC1 also caused a significant increase in the number of pHH3 positive cells as compared with that of control cells (CNE-1: 4.84%±0.37% in control group VS 15.97%±0.37% in DEPDC1 knockdown group; HNE-1: 3.06%±0.74% in control group VS 9.4%±0.54% in DEPDC1 knockdown group). Taken together, these results clearly demonstrated that DPEPC1 depletion mainly induces mitotic cell cycle arrest in NPC cells.

### DEPDC1 depletion causes mitotic defects in NPC cells

**Figure 5:**
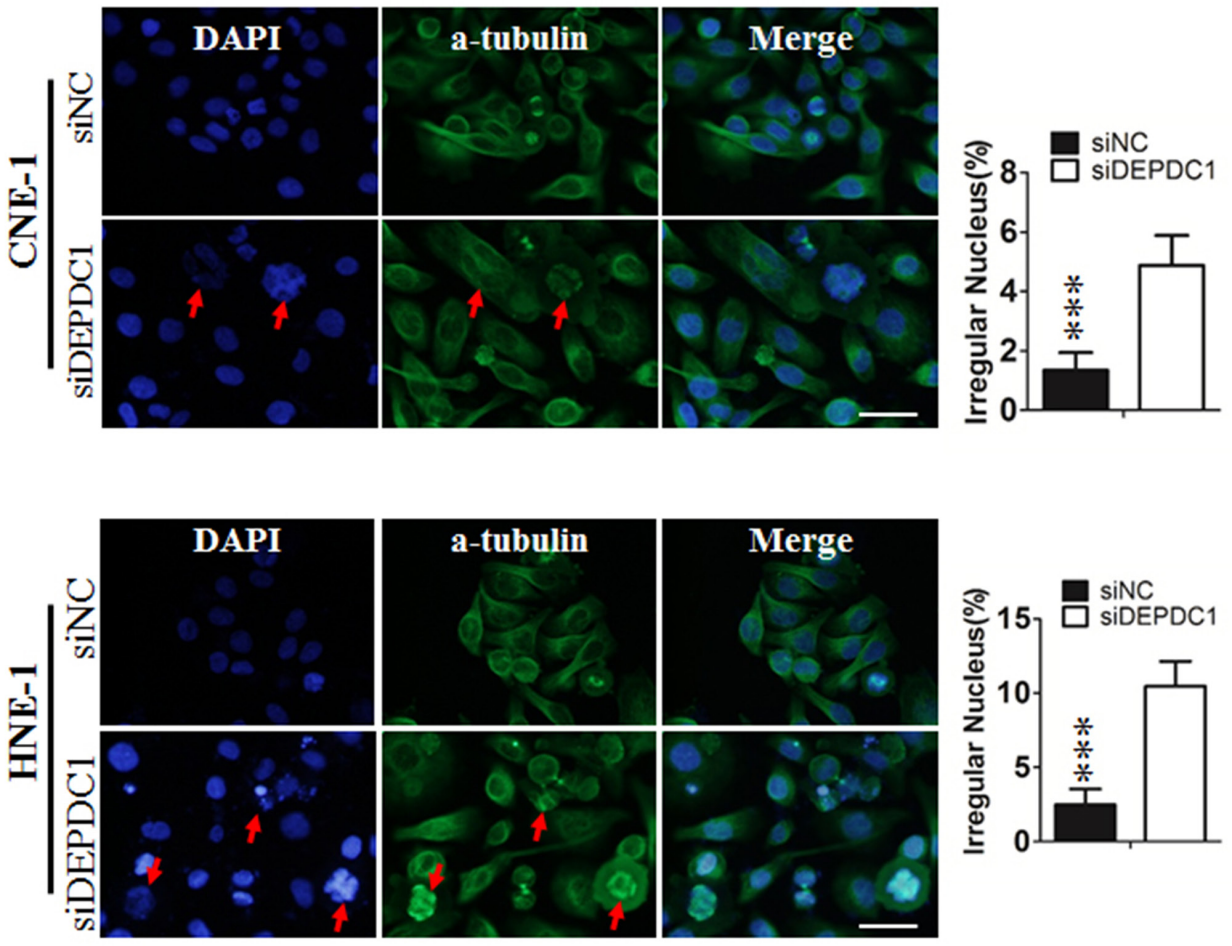
DEPDC1 depletion causes mitotic effects CNE-1 and HNE-1 cells were seeded and transfected with negative control siRNA or DEPDC1 siRNA. Forty-eight hours after transfection, cells were fixed and stained with anti-α-tubulin antibody (green). Representative images of cells with multipolar spindles and multinuclear structure (Red arrowheads indicated) were shown. The α-tubulin positive cells were counted and subjected to statistical analysis (P** < *0.01*, Student's t-test). Scale bar 20μm.

To determine whether DEPDC1 depletion causes mitotic defects in NPC cells, we performed indirect immunofluorescence staining with anti-α-tubulin antibody. The results showed that DEPDC1-depleted cells exhibited obvious mitotic defects including multipolar spindles and multiple nuclei in both cell lines (CNE-1: 1.02%±0.141% in negative control group VS 4.85%±0.25% in DEPDC1 knockdown group; HNE-1: 3.06%±0.74% negative control group VS 9.4%±0.54% DEPDC1 knockdown group), suggesting that DEPDC1 is essential for proper mitotic progression in NPC cells (Figure 5).

### DEPDC1 depletion induces apoptosis in NPC cells

**Figure 6:**
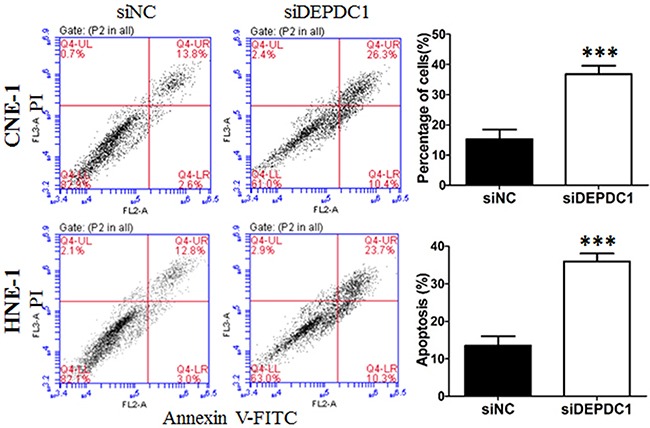
DEPDC1 depletion induces apoptosis CNE-1 and HNE-1 cells were transiently transfected with the control siRNA and DEPDC1 siRNA, respectively. Seventy-two hours after transfection, cells were collected, stained with Annexin V-FITC and propidium iodide (PI) and subjected to apoptosis analysis. The proportion of early apoptotic cells (FITC^+^/PI^−^, lower right) and late apoptotic and secondary necrotic cells (FITC^+^/PI^+^, upper right) were quantified and subjected to statistical analysis (P*** < *0.001*, Student's t-test).

To further determine whether DEPDC1 knockdown induces apoptosis in NPC cells, Annexin V-FITC and PI double staining with flow cytometry was conducted to detect the apoptotic cells. The results showed that, seventy-two hours after transfection, approximately 38% of DEPDC1 depleted CNE-1 cells displayed early apoptotic and late apoptotic/secondary necrotic phenotype, whereas only 17% of negative siRNA transfected cells had this phenotype (Figure 6). Similarly, DEPDC1 knockdown in HNE-1 cells also caused an increase in the apoptotic population as measured by Annexin V-FITC and PI double staining (33.4% in DEPDC1 knockdown group VS 14.07% in control group, Figure 6). These results suggested that DEPDC1 depletion induces apoptotic cell death.

### DEPDC1 depletion reduces migration and invasion ability in NPC cells

**Figure 7:**
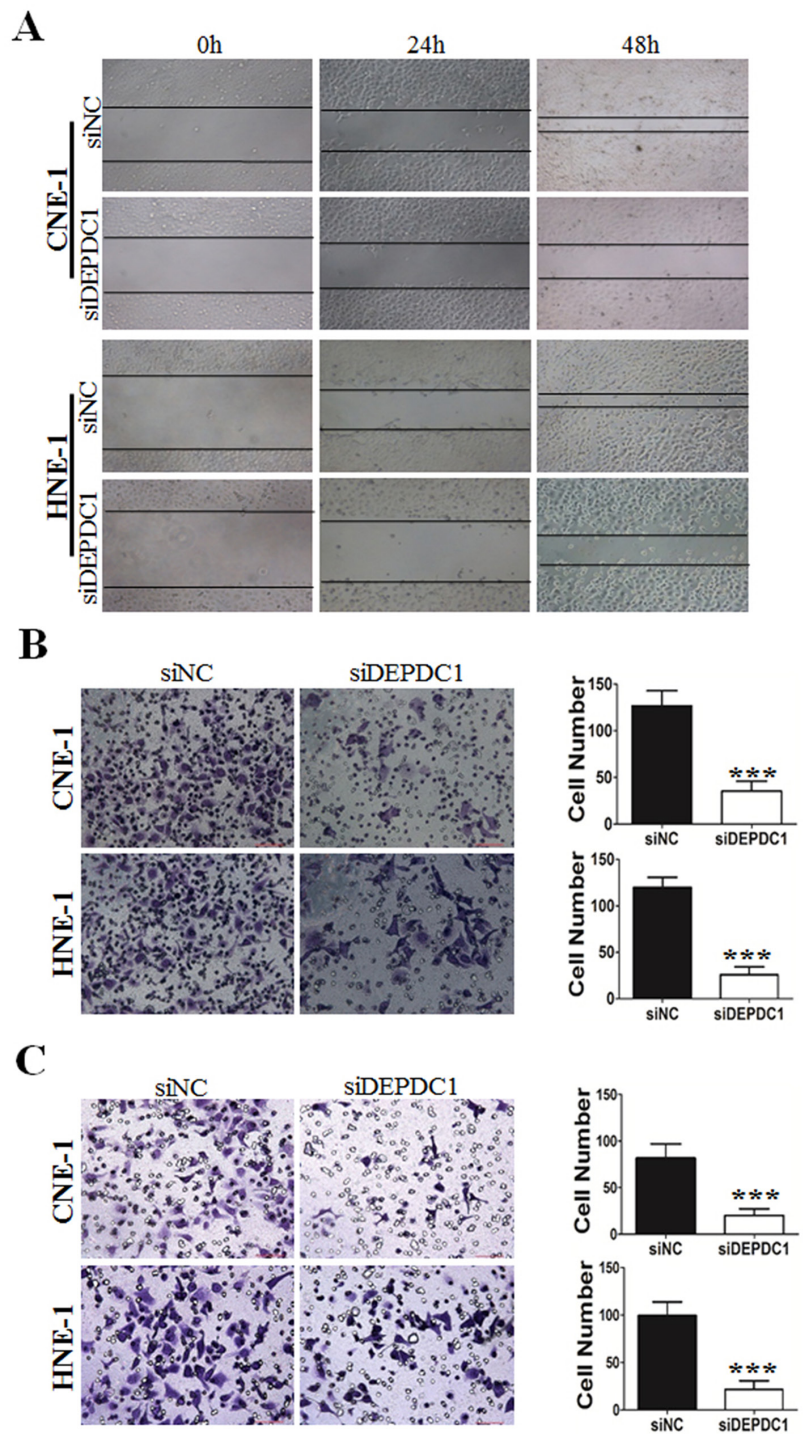
DEPDC1 depletion inhibits migration and invasion in NPC cells CNE-1 and HNE-1 cells were transiently transfected with control and DEPDC1 siRNA. Twenty-four hours after transfection, cells were subjected to would healing assay **(A)**, transwell migration **(B)** and transwell invasion **(C)** assay, respectively. Representative images were shown and the migrated and invaded cells were quantified and subjected to statistical analysis (P*** < *0.001*, Student's t-test).

Furthermore, we determine whether DEPDC1 depletionaffected cell migration and invasion ability in NPC cells (CNE-1 and HNE-1). Wound healing assay revealed that compared with control group, DEPDC1 depletion obviously decreased the rate of lateral migration into a wound introduced in a confluent monolayer of both HNE-1 and CNE-1 cells (Figure 7A). Besides, the cell migration and invasion ability was examined by transwell migration and invasion assay. As shown in Figure 7B, transwell migration assay showed that knockdown of DEPDC1 significantly inhibited the cellular migration ability compared with control group in both HNE-1 and CNE-1 cells. On the other hand, transwell invasion assay also showed that DEPDC1 depletion significantly decreased the invasion ability in both cell lines (Figure 7C).

### DEPDC1 depletion causes dysregulation of multiple downstream genes in NPC cells

**Figure 8:**
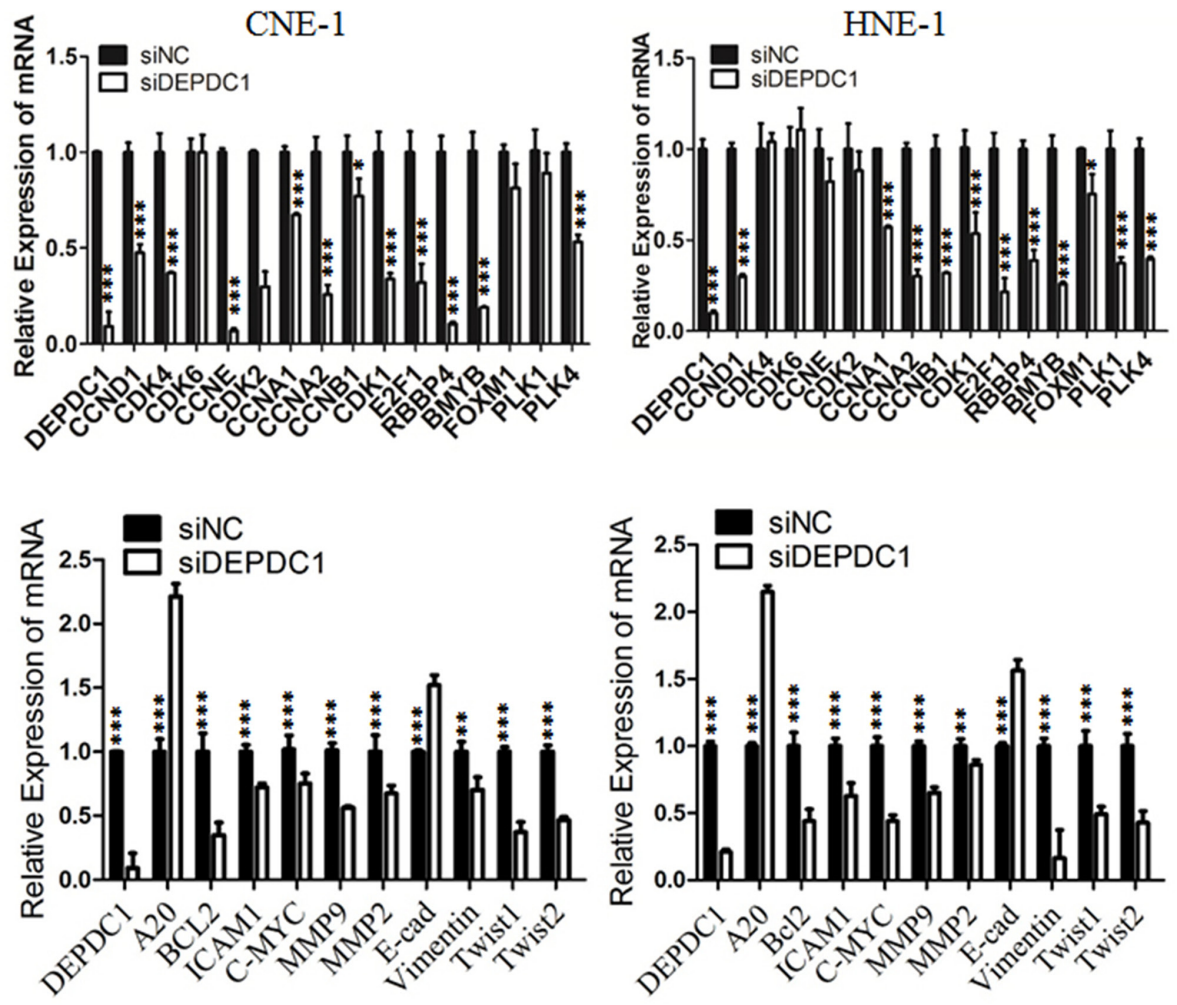
DEPDC1 depletion causes dysregulation of multiple downstream genes CNE-1 and HNE-1 cells were transiently transfected with control siRNA and DEPDC1 siRNA, respectively. Forty-eight hours after transfection, total RNA were prepared. Quantitative RT-PCR was used to determine the expression levels of indicated genes. GAPDH was used as an internal control.

We and others have demonstrated that DEPDC1 acts as a transcription repressor to inhibit the transcription of A20, a negative regulator of the NF-κB signaling pathway in bladder cancer cells and HeLa cells [8, 15]. It is well established that NF-κB plays an important role in cell proliferation, tumorigenesis and metastasis [22, 23]. Thus, we determined the transcription of A20 and several NF-κB regulated cell cycle genes upon DEPDC1 silencing in NPC cells. As shown in Figure 8, knockdown of DEPDC1 in both HNE-1 and CNE-1 cells caused significant upregulation of A20 and downregulation of mutiple NF-κB downstream target genes implicated in proliferation (c-Myc, BCL2, CCND1, CCNB1 and CCNB2), tumorigenesis (c-Myc,) and metastasis (MMP2, MMP9, ICAM1, vimentin, Twist1). Of note, other cell cycle related genes such as CCNA1, E2F1, RBBP4, B-Myb, and PLK4 were also significantly downregulated in DEPDC1 knockdown cells. In addition, silencing of DEPDC1 resulted into upregulation of E-cadherin (epithelial cell marker), and downregulation of vimentin (mesenchymal cell marker), Twist1 and Twist2 (transcription factors), suggesting that DEPDC1 might also regulate the process of epithelial-mesenchymal transition (EMT) [22, 23].

### DEPDC1 depletion inhibits NPC tumorigenesis in nude mice

**Figure 9:**
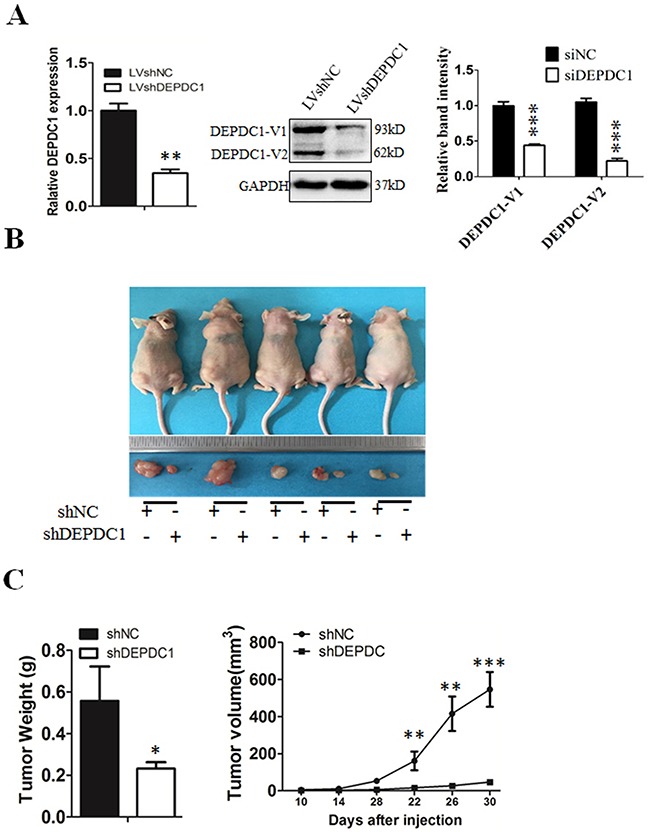
DEPDC1 depletion inhibits tumor growth *in vivo* **(A)** CNE-1 cells were infected with lentivirus particles expressing negative control and DEPDC1 shRNA, and then subjected to establish the negative control (shNC) and DEPDC1 stable knockdown (shDEPDC1) cells with puromycin selection. Quantitative RT-PCR and immunoblotting were used to confirm the stable knockdown effect of DEPDC1 expression. Optical densities (O.D.) of individual bands on immunoblots were quantified as described in Figure 2B. **(B)** The stable control cells (shNC) and DEPDC1 knockdown cells (shDEPDC1) were injected subcutaneously into the right and left dorsal flank of nude mice, respectively. The mice were sacrificed thirty days after injection. **(C)** The tumor weight was measured at the end of the experiment, and the tumor size was measured about twice a week for tumor growth curve construction. (P* < *0.05*, P** < *0.01*, P** < 0.001).

To finally determine whether silencing of DEPDC1 expression could inhibit the NPC tumorigenicity *in vivo*, we used CNE-1 to establish a DEPDC1 knockdown stable cell line. Quantitative RT-PCR and Western blot analysis demonstrated that the DEPDC1 expression was significantly silenced at both mRNA and protein levels in the stable DEPDC1 knockdown cells (Figure 9A). Consistent with the phenotypes observed in transient knockdown experiments (Figures 2, 3, 4, 5, 6, 7), stable knockdown of DEPDC1 also caused a significant inhibition of cell proliferation and mitotic arrest (Supplementary Figure 1A and 1B). Cell migration and invasion assays showed that stable knockdown of DEPDC1 also significantly inhibited the cellular migration and invasion ability (Supplementary Figure 2A and 2B). Subsequently, the expression of downstream genes such as CCNA1 and vimentin were also significantly downregulated upon stable silencing of DEPDC1 expression (Supplementary Figure 3A and 3B). Furthermore, the stable DEPDC1 knockdown and the control cells were injected subcutaneously into the right and left dorsal flank of nude mice, respectively. The results indicated that the tumor formation rate in LV-shNC group was 100%, and in LV-shDEPDC1 group was 60% (Figure 9B). Of note, knockdown of DEPDC1 in CNE-1 stable cell lines caused an obvious decrease in tumor weight and volume compared with the control group (Figure 9C). At the end of the study (day 30), tumor weight and tumor size of DEPDC1 knockdown group (0.23±0.03g, 46.91±15.07mm^3^) was only 41.1% and 8.6% of the control group (0.56±0.17g, 546.24±93.46mm^3^) (Figure 9B and 9C).

## DISCUSSION

To date, several studies have shown that DEPDC1 is aberrantly overexpressed in several types of cancers suggesting its therapeutic potential in cancers [8, 9, 12–14]. This study is an effort to investigate whether DEPDC1 could be exploited as a novel therapeutic target for the treatment of NPC. We have found that the expression of DEPDC1 is significantly upregulated in NPC samples relative to non-tumor tissues, and siRNA mediated depletion of DEPDC1 leads to a significant decrease in cell viability and motility, cell cycle arrest and mitotic defects followed by apoptotic cell death in both two representative NPC cell lines studied. Knockdown of DEPDC1 also significantly inhibits tumor growth in vivo. These results strongly indicated that, apart from being of diagnostic value, inhibition of DEPDC1 may additionally serve to be of therapeutic value in NPC. Notably, a recently developed cell-permeable peptide against DEPDC1 achieved good therapeutic effects in bladder cancer. Thus, it is necessary to determine the peptide's therapeutic effects in NPC in the future study.

Currently, functional analysis on the implication of DEPDC1 in cancer development has been conducted in bladder cancer, multiple myoloma and prostate cancer [8, 9, 12, 14]. It has been shown that knockdown of endogenous DEPDC1 expression by siRNA resulted in significantly growth suppression in these cancer cell lines. Another study also showed that DEPDC1 is associated with the antitumor activity of MELK (maternal embryonic leucine zipper kinase) inhibitor in breast cancer cells [24]. In consistent with these findings, our present study demonstrated that knockdown of DEPDC1 significantly inhibited the growth of NPC cells both *in vitro* and *in vivo* (Figure 2). Of note, we further showed that knockdown of DEPDC1 reduces the migration and invasion ability in NPC cells suggesting that overexpression of DEPDC1 is required for the accelerated proliferation and motility in NPC cells. Previous reports showed that DEPDC1 interacts with ZNF224 to repress the transcription of A20, resulting in the activation of the NF-κB pathway in bladder cancer [8]. Consistent with this report, we found that DEPDC1 knockdown resulted in significant upregulation of A20 as well as several NF-κB regulated genes in NPC cells. It is well known that NF-κB pathway plays a critical role in tumorgenesis and metastasis as well as inflammatory diseases, and serves as an effective target for the treatment of various cancers [22, 23, 25]. Thus, further studies are needed to investigate whether rational combination of DEPDC1 siRNA or inhibitors with the IKK/NF-κB inhibitors or chemotherapeutic agents will achieve a synergistic antitumor effect in NPC as well as other types of cancers. Our preliminary data with limited number of tissues also revealed that DEPDC1 seems to be overexpressed in inflammatory tissues as well as NPC tissues (Supplementary Table 1). NF-κB pathway has been shown to be constitutively activated in NPC cells by either genetic changes or EBV latent genes [26, 27]. Extracellular inflammatory stimuli also trigger the activation of NF-κB signaling to facilitate NPC progression and development (Kan 2015; Chung 2013). Thus, it is worthwhile to further investigate the functional implication of DEPDC1-mediated activation of NF-κB pathway in tumor-derived inflammation as well as NPC development.

In addition, previous reports showed that although DEPDC1 knockdown resulted in obvious loss-of-function phenotype, introduction of DEPDC1 expression construct into NIH3T3 cells could not enhance the growth of cells, and DEPDC1 overexpression caused no enhancement of cell migration and invasion potentials in bladder cancer cells [8, 9]. In line with these reports, we also introduced DEPDC1 expression constructs into NPC cells and found no obvious changes of cell growth caused by DEPDC1 overexpression (data not shown). Taken together, these data suggest that DEPDC1 alone is unable to drive the accelerated proliferation and motility of malignant cells, and probably need the cooperation and interaction with other proteins. To date, only one protein, ZNF224, has been identified to form a complex with DEPDC1 in bladder cancer cells [8, 9]. However, studies indicated that the proliferation-promoting effect caused by ZNF224 and DEPDC1 double overexpression is not robust compared with single ZNF224 or DEPDC1 overexpression [8]. Thus, further studies are required to identify more other potential proteins which interact with DEPDC1.

Several studies suggested that overexpression of DEPDC1 in cancers might be regulated through multiple molecular mechanisms. Ramalho-Carvalho and colleagues reported that miR-130a represses the expression of DEPDC1, and epigenetic disruption of miR-130a causes up-regulation of DEPDC1 in prostate cancer [14]. Intriguingly, Chung and colleagues showed that DEPDC1 is a downstream molecule of the MELK signaling pathway, and MELK enhances DEPDC1 phosphorylation and its stability [24]. As up-regulation of DEPDC1 mRNA has been observed in NPC as well as other cancers, it is highly likely that DEPDC1 could be also aberrantly up-regulated at transcriptional level in cancers. It is also worthwhile to mention that DEPDC1 is located at the region of 1p31.3 that shows copy number amplification in breast cancer [28]. Thus, further studies are also needed to determine the molecular mechanisms of DEPDC1 up-regulation in NPC.

## MATERIALS AND METHODS

### Cell culture

Two representative NPC cell lines, namely CNE-1 and HNE-1, with different differentiation and EBNA (Epstein-Barr virus nuclear antigen) status were used in this study. Both are squamous cells. HNE-1 is poorly differentiated and EBNA positive whereas CNE-1 is well differentiated and EBNA negative. Both cell lines were cultured in RPMI 1640 medium supplemented with 100 units/ml penicillin, 100mg/ml streptomycin, and 10% fetal bovine serum (FBS) (Invitrogen), and maintained at a humidified incubator containing 5% CO_2_ at 37°C. Cells were routinely tested for myoplasma.

For transfection, cells were seeded at a density of 5 × 10^4^ cells/24-well tissue culture plate or 2 × 10^5^ cells/6-well tissue culture plate and allowed to attach overnight.

### Tissue specimens

All human tissue specimens were obtained from April 2013 to December 2015 at the Department of Otolaryngology, The First Affiliated Hospital of Chongqing Medical University. Totally, a collection of 33 NPC and 18 unpaired normal nasopharyngeal tissues were snap-frozen and preserved in liquid nitrogen until use. None of the patients have received radiation treatment before. All human tissue was collected according to protocols approved by the Ethics Committee of the First Affiliated Hospital of Chongqing Medical University. Written consent was obtained from all subjects. Studies using human material were approved by the First Affiliated Hospital of Chongqing Medical University.

### Tissue microarrays and immunohistochemistry

DEPDC1 protein expression was determined on a NPC tissue microarray slide from Auragene (Cat. no. TC0075, Hunan China). The NPC tissue microarray TC0075 contains human nasopharyngeal cancer (44 cases), papilloma (30 cases), polyp (12 cases), chronic hyperplasia (6 cases), chronic inflammation (6 cases) and adjacent normal nasal tissues (2 cases). The information of tissue microarray TC0075 was provided in Supplementary Table 2. For immunohistochemistry, slides were routinely deparaffinized in xylene and rehydrated in a graded series of alcohol solutions, and then were subjected to Heat-induced antigen retrieval in EDTA (1 mM, pH 8.0). 3% hydrogen peroxide was then used to blocked Endogenous peroxidase activity for 15 min. Then the slides were incubated with DEPDC1 rabbit polyclonal antibody at 4°C for overnight. The information of the antibodies used were listed in Supplementary Table 4. Detection was carried out using Universal Immuno-peroxidase Polymer Anti-Mouse/Rabbit Immunohistochemical Staining Reagent (Cat. no. PV-8000; ZSGB-Bio, Beijing, China). Diaminobenzidine (Cat. no. DAB-P013IH; Auragene, Hunan, China) was used for color development, and hematoxylin was used for counterstain. Negative control was carried out using normal rabbit IgG. According to the previous studies [19], the expression of DEPDC1 was evaluated by the intensity and extent of the staining. In brief, the intensity was scored as negative=0, weak=1, moderate=2, or strong=3,and the extend was scored as 0%=0, 1-25%=1, 26-50%=2, 51-75%=3, or 76-100%=4. The final score was determined by the sum of the intensity and extent scores (0-7). For analysis of DEPDC1 expression, tumors having a final score of <3 were considered as low expression and those with scores ≥3 as high expression.

### siRNA synthesis and transfection

The negative control siRNA, and siRNAs against DEPDC1 were chemically synthesized by Shanghai Sangon Biotech (Shanghai, China). The sequences of the siRNAs used were listed in Supplementary Table 3. The siRNAs were transfected into the NPC cells at a final concentration of 10 nmol/L using Lipofectamine RNAiMAX reagent (invitrogen) according to the manufacturer's instructions. Cells were collected and subjected to subsequent analysis 24h to 72h after transfection.

### RNA isolation and RT-PCR

Total RNA isolated from fresh frozen tissues and NPC cell using the Total RNA Kit I (Omega Bio-Tek) according to the manufacturer's instructions. Quantitative RT-PCR were conducted as described previously [20]. The sequences of the primers used can be found in Supplementary Table 3.

### Immunoblotting analysis

The immunoblotting analysis was conducted as described previously [20]. Briefly, cells were collected and lysed in RIPA buffer, supplemented with protease inhibitor PMSF (Beyotime, Jiangsu, China). The total proteins were quantified using the bicinchoninic acid (BCA) protein assay kit (Thermo Scientific, Beijing, China). The optical density of protein bands after immunoblotting was quantified using Quantity One software (Bio-Rad). The information of the antibodies used were were listed in Supplementary Table 4. The blots were visualized by enhanced chemiluminescence (ECL, Amersham).

### Cell proliferation assays

Cell proliferation was measured using Cell Counting Kit-8 (Dojindo, Tokyo, Japan). Briefly, 1000 cells/well were plated into 96-well plates. At the indicated time points (0h, 24h, 48h, 72h), cells were treated with 10μl of the CCK8 solution for 1h at 37°C. The absorption at 450 nm was measured for cell survival rate calculation. All assays were performed in triplicates.

### Cell cycle and apoptosis analysis

Cells were collected by trypsin digestion and low speed centrifugation (1000 rpm for 5min), and then subjected to cell cycle analysis on a FACScan flowcytometer as described previously [21]. For apoptosis analysis, cells were stained with Annexin V-FITC and propidium iodide using the Annexin V-FITC Apoptosis Detection Kit (Sigma, Saint Louis, USA), and then subjected to FACS analysis.

### Indirect immunofluorescent assays

The indirect immunofluorescent assays were conducted as described previously with minor modifications [15]. Briefly, CNE-1 and HNE-1 cells were seeded on glass coverslips in 24-well plates. Cells were fixed in freshly prepared ice-cold 4% formaldehyde for 20 min at room temperature, permeabilized with 1% Triton for 15 min and blocked with 5% bovine serum albumin (BSA) at room temperature for 2 h. After washing with PBS, cells were incubated with primary antibodies at 4°C for overnight. The next day, after washing with PBS, cells were incubated with the corresponding secondary antibodies at 37°C for 1 h. The nucleus was stained with 4′6-diamidino-2-phenylindole (DAPI, Vector Laboratories) for 3 min. and cells were visualized with an Olympus BX51 fluorescence microscope and a Zeiss confocal LSM 768 microscope. For BrdU labelling, cells were treated with BrdU for 30min, fixed with 70% ethanol at 4°C, and the rest steps were the same as the indirect immunofluorescence assay. The antibodies used were listed in Supplementary Table 4.

### Migration and invasion assays

Would healing assays, and the transwell migration and invasionassays were performed as described previously [20].

### Stable knockdown cell line generation

A lentivirus-based RNAi approach was used to generate the stable DEPDC11 knockdown cell line. Based on the efficient siRNA sequences used in transient experiment, the corresponding double-stranded short hairpin RNA (shRNA) template for both the negative control and DEPDC1 were designed and cloned into the Hind III/Bgl II sites of the lentiviral vector (Shanghai Genechem, Shanghai, China), respectively. The lentivirus particles were packaged and prepared by cotransfection of the lentiviral vectors and pHelper vectors (Shanghai Genechem) into 293T cells by Lipofectamine 2000 (Invitrogen), followed by routine culture supernatant collection and concentration. All lentiviral vectors expresses GFP, which facilitates the monitoring of transfection efficiency and selection of stable cell lines. The lentivirus particles carrying negative control and DEPDC1 shRNA were used to infect CNE-1 cells. Forty-eight hours after infection, cells were selected in the presence of puromycin (CNE-1 1.5μg/μl) for about two weeks to generate the negative control and DEPDC1 stable knockdown cells, designated as shNC and shDEPDC1, respectively.

### Tumor xenografts

6×10^6^ stable DEPDC1 knockdown CNE-1 cells (shDEPDC1) and control cells (shNC) were subcutaneously injected into the right and left dorsal flank of four to six-week old nude mice (BALB/c), respectively. The tumor size was measured every 4 days by a vernier caliper along two perpendicular axes. The volume of the tumor was calculated following the formula: volume= 1/2×length×width^2^. About 30 days after injection, the mice were killed, and the tumor specimens were weighed and fixed as described previously [20]. All experimental procedures involving animals were performed in accordance with animal protocols approved by Laboratory Animal Center of Chongqing Medical University.

### Statistical analysis

All statistical analysis were carried out using the SPSS 16.0 statistical software package (SPSS Inc., Chicago, USA). Student's t-test and Mann-Whitney test were used. The distribution of data were expressed as mean±SD. P < 0.05 was considered statistically significant.

## SUPPLEMENTARY FIGURES AND TABLES









## Abbreviations

DEPDC1: DEP domain containing 1
NPC: nasopharyngeal carcinoma
EBNA: Epstein-Barr virus nuclear antigen
FBS: fetal bovine serum
BCA: bicinchoninic acid
