# Supplementary material for: DEPDC1 is required for cell cycle progression and motility in nasopharyngeal carcinoma
Source: Oncotarget. 2017 Jun 29;8(38):63605–19. doi: 10.18632/oncotarget.18868 (PMC5609947; doi:10.18632/oncotarget.18868)
Supplement: Supplementary file 1 [file oncotarget-08-63605-s001.pdf]

DEPDC1 is required for cell cycle progression and motility in nasopharyngeal carcinoma

SUPPLEMENTARY MATERIALS

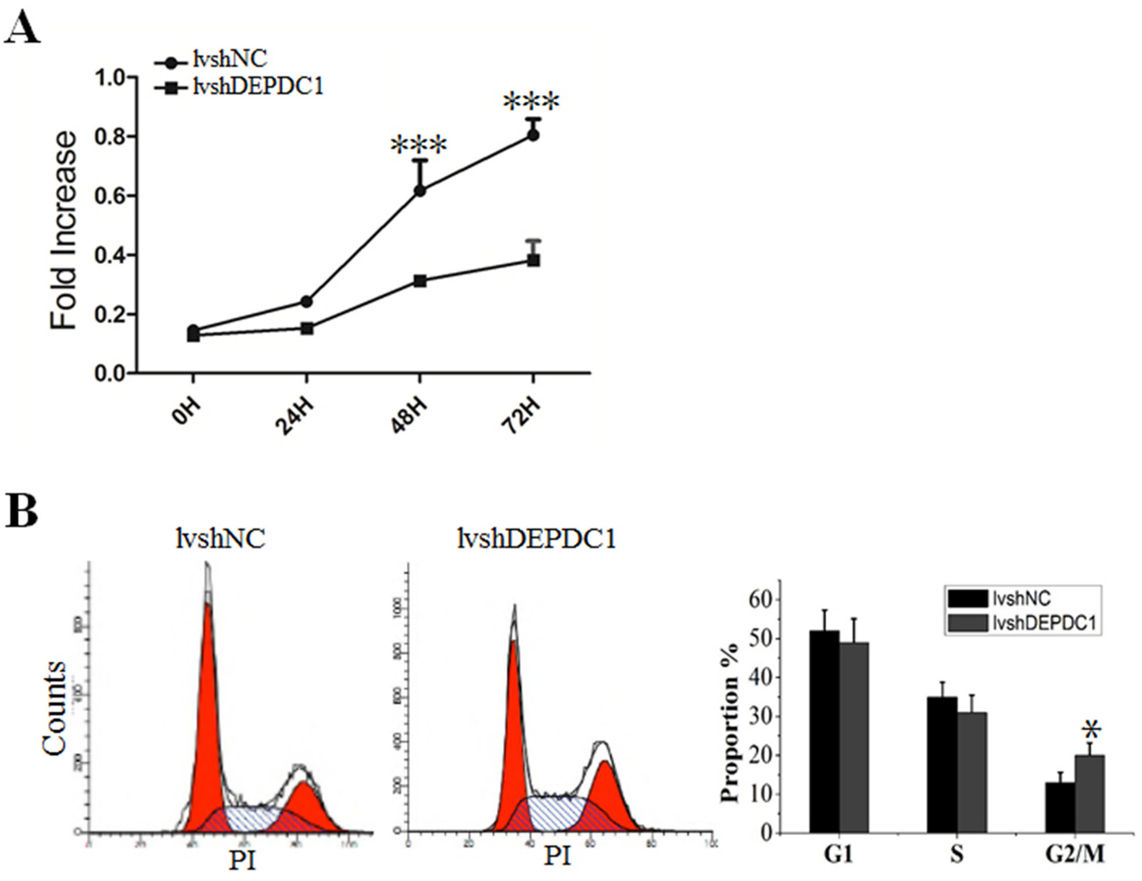

Supplementary Figure 1: DEPDC1 stable knockdown inhibits proliferation and cell cycle progression.

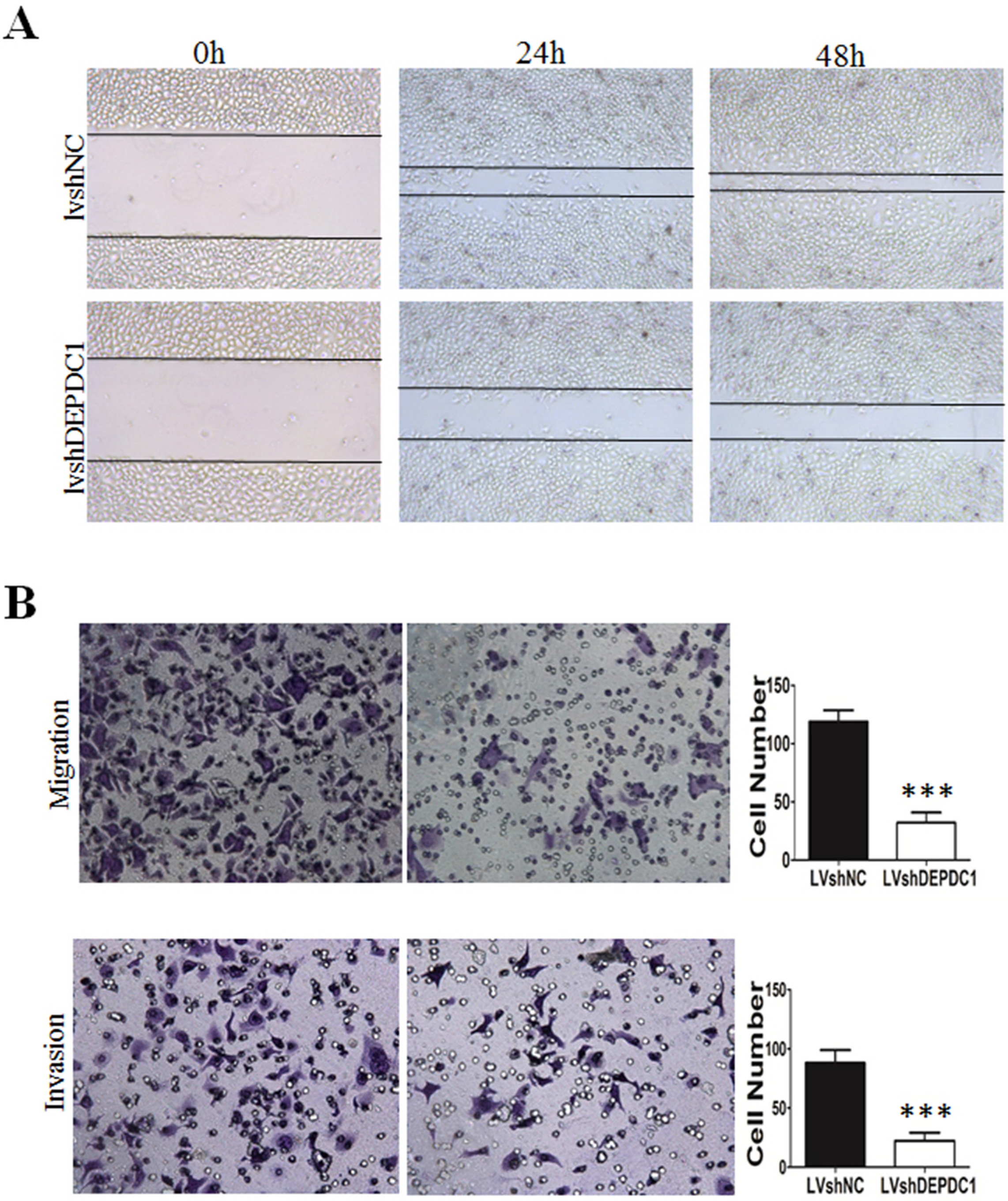

Supplementary Figure 2: DEPDC1 stable knockdown reduces migration and invasion.

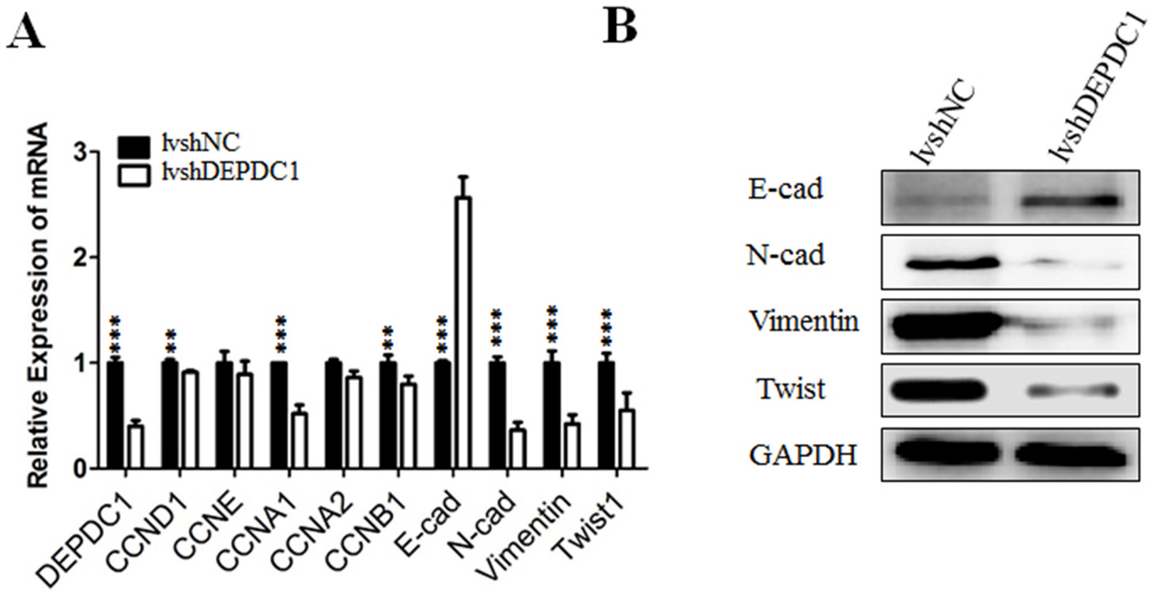

Supplementary Figure 3: DEPDC1 stable knockdown causes dysregulation of multiple downstream genes.

Supplementary Table 1: Correlation of clinicopathologic parameters with DEPDC1 expression

Pathological variables	Sample no.	DEPDC1 IHC staining (%)		P value
		Negative	Positive	
Pathological types				
Normal nasal tissues	2	2(100)	0(0)	
Polyp	12	12(100)	0(0)	
Chronic hyperplasia	6	4(66.7)	2(33.3)	
Chronic inflammation	6	2(33.3)	4(66.7)	
Papilloma	30	5(16.7)	25(83.3)	
NPC tissues	44	18(41)	26(59)	
NPC vs Non tumor†				
Non-tumor tissues	26	19 (73)	7 (27)	0.018*
NPC tissues	44	18(41)	26(59)	
Age(years)				
<60	72	32(44.4)	40(55.6)	0.810
≥60	28	10(35.7)	18(64.3)	
Sex				
Male	80	48(60)	32(40)	0.382
Female	20	9(45)	11(55)	
Tumor size				
T1	6	0(0)	6(100)	0.336
T2	16	7(43.8)	9(56.2)	
T3	6	4(66.7)	2(33.3)	

†Non-tumor tissues include normal nasal tissues, polyp, chronic hyperplasia, chronic inflammation.

Supplementary Table 2: NPC tissue microarray information (No. TC0075).

See Supplemenetary File 1

Supplementary Table 3: Oligonucleotides used in the present study.

See Supplemenetary File 2

Supplementary Table 4: Antibodies used in the present study.

See Supplemenetary File 3
