# Supplementary material for: DEPDC1 is required for cell cycle progression and motility in nasopharyngeal carcinoma
Source: Oncotarget. 2017 Jun 29;8(38):63605–19. doi: 10.18632/oncotarget.18868 (PMC5609947; doi:10.18632/oncotarget.18868)
Supplement: Supplementary file 2 [file oncotarget-08-63605-s002.docx]

**Supplementary Table 2: NPC tissue microarray information (No. TC0075)**

| **#** | **Pos** | | **Sx** | | **Age** | **Tissue** | **Pathological Diagnosis** | **Grade** | | **Score** | |
| --- | --- | --- | --- | --- | --- | --- | --- | --- | --- | --- | --- |
| 1 | | 1A | | M | 71 | Nose | Squamous cell carcinoma | | 1 | | 4 |
| 2 | | 1B | | M | 71 | Nose | Squamous cell carcinoma | | 1 | | 4 |
| 3 | | 1C | | M | 66 | Nose | Squamous cell carcinoma | | 1 | | 3 |
| 4 | | 1D | | M | 66 | Nose | Squamous cell carcinoma | | 1 | | 5 |
| 5 | | 1E | | M | 46 | Nose | Squamous cell carcinoma | | 1 | | 4 |
| 6 | | 1F | | M | 46 | Nose | Squamous cell carcinoma | | 1 | | 3 |
| 7 | | 1G | | M | 77 | Nose | Squamous cell carcinoma | | 2 | | 0 |
| 8 | | 1H | | M | 77 | Nose | Squamous cell carcinoma | | 2 | | 0 |
| 9  10  11  12  13  14  15  16  17  18  19  20  21  22  23  24  25  26  27  28  29  30  31  32  33  34  35  36  37  38  39  40  41  42  43  44  45  46  47  48  49  50  51  52  53  54  55  56  57  58  59  60  61  62  63  64  65  66  67  68  69  70  71  72  73  74  75  76  77  78  79  80  81  82  83  84  85  86  87  88  89  90  91  92  93  94  95  96  97  98  99  100 | | 1I  1J  2A  2B  2C  2D  2E  2F  2G  2H  2I  2J  3A  3B  3C  3D  3E  3F  3G  3H  3I  3J  4A  4B  4C  4D  4E  4F  4G  4H  4I  4J  5A  5B  5C  5D  5E  5F  5G  5H  5I  5J  6A  6B  6C  6D  6E  6F  6G  6F  6I  6J  7A  7B  7C  7D  7E  7F  7G  7H  7I  7J  8A  8B  8C  8D  8E  8F  8G  8H  8I  8J  9A  9B  9C  9D  9E  9F  9G  9H  9I  9J  10A  10B  10C  10D  10E  10F  10G  10H  10I  10J | | M  M  M  M  M  M  M  M  F  F  M  M  M  M  M  M  M  M  F  F  M  M  F  F  F  F  M  M  F  F  M  M  M  M  M  M  M  M  M  M  M  M  M  M  M  M  F  F  M  M  M  M  M  M  M  M  M  M  F  F  F  F  M  M  M  M  M  M  M  M  M  M  M  M  M  M  M  M  M  M  F  F  F  F  M  M  M  M  M  M  M  M | 60  60  36  36  48  48  59  59  41  41  40  40  45  45  50  50  32  32  49  49  54  54  53  53  79  79  62  62  70  70  57  57  73  73  45  45  46  46  27  27  41  41  50  50  76  76  71  71  52  52  51  51  46  46  59  59  56  56  40  40  68  68  57  57  31  31  46  46  18  18  20  20  65  65  72  72  39  39  45  45  47  47  16  16  37  37  42  42  62  62  41  41   \| 5436 \| \| --- \| | Nose  Nose  Nasopharynx  Nasopharynx  Nose  Nose  Nose  Nose  Nose  Nose  Nose  Nose  Nose  Nose  Nose  Nose  Nose  Nose  Nose  Nose  Nose  Nose  Nose  Nose  Nose  Nose  Nose  Nose  Nose  Nose  Nose  Nose  Nose  Nose  Nose  Nose  Nose  Nose  Nose  Nose  Nose  Nose  Nose  Nose  Nose  Nose  Nose  Nose  Nose  Nose  Nose  Nose  Nose  Nose  Nose  Nose  Nose  Nose  Nose  Nose  Nose  Nose  Nose  Nose  Nose  Nose  Nose  Nose  Nose  Nose  Nose  Nose  Nose  Nose  Nose  Nose  Nose  Nose  Nose  Nose  Nose  Nose  Nose  Nose  Nose  Nose  Nose  Nose  Nose  Nose  Nose  Nose | Squamous cell carcinoma  Squamous cell carcinoma  Squamous cell carcinoma  Squamous cell carcinoma  Squamous cell carcinoma  Squamous cell carcinoma  Squamous cell carcinoma  Squamous cell carcinoma  Squamous cell carcinoma  Squamous cell carcinoma  Squamous cell carcinoma  Squamous cell carcinoma  Squamous cell carcinoma  Squamous cell carcinoma  Squamous cell carcinoma  Squamous cell carcinoma  Squamous cell carcinoma  Squamous cell carcinoma  Squamous cell carcinoma  Squamous cell carcinoma  Squamous cell carcinoma  Squamous cell carcinoma  Basal cell carcinoma  Basal cell carcinoma  Basal cell carcinoma  Basal cell carcinoma  Basal cell carcinoma  Basal cell carcinoma  Adenoid cystic carcinoma  Adenoid cystic carcinoma  Adenocarcinoma  Adenocarcinoma  Undifferentiated carcinoma  Undifferentiated carcinoma  Undifferentiated carcinoma  Undifferentiated carcinoma  Inverted papilloma  Inverted papilloma  Inverted papilloma  Inverted papilloma  Inverted papilloma  Inverted papilloma  Inverted papilloma  Inverted papilloma  Inverted papilloma  Inverted papilloma  Inverted papilloma  Inverted papilloma  Inverted papilloma  Inverted papilloma  Inverted papilloma  Inverted papilloma  Inverted papilloma  Inverted papilloma  Inverted papilloma  Inverted papilloma  Inverted papilloma  Inverted papilloma  Inverted papilloma  Inverted papilloma  Inverted papilloma  Inverted papilloma  Inverted papilloma  Inverted papilloma  Inverted papilloma  Inverted papilloma  Polyp (chronic inflammation)  Polyp (chronic inflammation)  Polyp  Polyp  Polyp  Polyp  Polyp  Polyp  Polyp  Polyp  Polyp  Polyp  Chronic inflammation of mucosa with hyperplasia of No. 19  Chronic inflammation of mucosa with hyperplasia of No. 19  Chronic inflammation of mucosa with hyperplasia Chronic inflammation of mucosa with hyperplasia  Chronic inflammation of mucosa with hyperplasia  Chronic inflammation of mucosa with hyperplasia  Chronic inflammation  Chronic inflammation  Chronic inflammation  Chronic inflammation  Chronic inflammation  Chronic inflammation  Cancer adjacent normal  nasal tissue  Cancer adjacent normal  nasal tissue | | -  -  2  2  2  2  2  2  2  2  2  2  2  2  2  2  3  3  3  3  3  2-3  -  -  -  -  -  -  -  -  3  -  -  -  -  -  -  -  -  -  -  -  -  -  -  -  -  -  -  -  -  -  -  -  -  -  -  -  -  -  -  -  -  -  -  -  -  -  -  -  -  -  -  -  -  -  -  -  -  -  -  -  -  -  -  -  -  -  -  -  -  - | | 0  0  5  5  0  0  2  0  0  4  5  3  5  4  4  4  3  5  0  0  0  0  0  0  3  5  4  3  3  2  2  2  3  3  5  3  2  4  4  2  4  4  5  4  3  3  5  3  4  5  3  3  5  5  4  3  3  3  4  4  3  5  1  3  2  1  0  1  0  0  1  0  0  2  1  0  0  1  4  4  0  0  0  0  3  3  0  3  4  2  2  3   \| 5 \| \| --- \| |
