# Supplementary material for: DEPDC1 is required for cell cycle progression and motility in nasopharyngeal carcinoma
Source: Oncotarget. 2017 Jun 29;8(38):63605–19. doi: 10.18632/oncotarget.18868 (PMC5609947; doi:10.18632/oncotarget.18868)
Supplement: Supplementary file 3 [file oncotarget-08-63605-s003.docx]

**Supplementary Table 3: Oligonucleotides used in the present study**

**Primers used for RT-PCR analysis**

| **Gene name** |  | **Primer sequence** |  |
| --- | --- | --- | --- |
| GAPDH |  | F :5'-ACCTGACCTGCCGTCTAGAA-3'  R: 5'-TCCACCACCCTGTTGCTGTA-3'  F: 5'-GGTGTGCCATCCCTAGAAGA-3'，  R: 5'-CATTGCTTCTTGGCCAATTT-3'  F: 5’-TGAAGGTGACAGAGCCTCTGGAT-3’  R: 5’-TGGGTGAATTCGGGCTTGTT-3’  F:5’-CCAAACTTTTCCTCCCTGAACC-3’  R: 5'-GTGATGCTGAGAAGTTTCGTTGA-3  F: 5'–GCCTTCTCGGTCTGGAGGAT-3'  R:5'-TTTCTCCTTCTCTGGAAACAATGAC-3  F: 5’-TCTCCGTGATTGCTTGGCTA -3’  R: 5’-AGCAGGATACACAGCCACAC-3’  F:5′-ATGCACCGATACACACTGGA-3′  R: 5′-CACAAGCTTCCGGACTTCTC-3′  F:5′-TTCGGGTAGTGGAAAACCAG-3′  R: 5′-CAGCAGCTCGAATTTCTTCC-3′  F:5′-CAGAGGTTGAACCCCACAGT-3′  R: 5′-CCTCTGGCTTCGTCAGAATC-3′  F:5′-TTGACAGCGACAAGAAGTGG-3′  R: 5′-TCACGTCGTCCTTATGCAAG -3′  F:5′-GGATGCCTTTGTGGAACTGT-3′  R: 5′-AGC CTG CAG CTT TGT TTC AT-3′  F:5′-GCTATGGACCTTGGGAGAA-3′  R: 5′-GCTATGGACCTTGGGAGAA-3′  F: 5'-GCATGTTCGTGGCCTCTAAG-3′  R: 5'-CGTGTTTGCGGATGATCTGT-3′  F: 5'-TTGCATCGTTCACCGAGATC-3′  R: 5'-CTGGTAGCTGTAGATTCTGGCCA-3′  F: 5'-CGTGGTCAGGTTGTTTGATG-3′  R: 5'-CGGGCTCTGGAACTTTATCC-3′  F: 5'-CTCCAGGAAGAGGAAGGCAA-3′  R: 5'-TCGATTTTGGCCATTTCTTCA-3′  F: 5'-TTTTGGAGTCCCTGTTCG-3'  R: 5'-CCAGGCTCCAGATGTCCA-3'  F: 5'-ACCCCAAGAGTGGAGTTGTG-3'  R: 5'-GGAAGGCATTTTCTGATCCA-3'  F: 5'-GAGCGCTTTCATTGGTCCAT-3'  R: 5'-GACCCGGCCAAAGAATAGTC-3'  F: 5'-GGTGTGCCATCCCTAGAAGA-3′  R: 5'-CATTGCTTCTTGGCCAATTT-3′  F: 5'-TGCAGGATTCCAGGTTAT-3'  R: 5'-GTACTGACCAGGAGGGAT-3'  F: 5'-CCATCAGTACCTGGCCGAGAGC-3'  R: 5'-CGCTTCTGCACCTTCAGCACCT-3'  F:5'-AGACTTGCGTCTCCGTGGACAT-3'  R:5'-CCTCCTTTGGAACGGCACTGAT-3'  F: 5'-AGAAACGAGCCTGCCTGCCTTACA-3'  R: 5'-AGATGGTTCCTCAGGGAGGT-3'  F: 5'-AAACCTGCAGCTAGGGATGT-3'  R: 5'-AGCCCAGTCCATCAGAACTC-3'  F: 5'-GGCAACCTTTTCCTGAATGA-3'  R: 5'-AATGGACCACACATCCACCT-3'  F: 5' -TGGTTTCAGCGTCGTCGC -3'  R: 5' -ATTAGCTTCCTTCACGCAGT-3' | |
| DEPDC1  E-cadherin |  |  |  |
| Vimentin  Twist1  Twist2  A20  C-myc  ICAM  MMP9  BCL-2  MMP2  CCND1  CDK4  CDK6  CCNE  CDK2  CCNA1  CCNA2  CCNB1  CDK1  E2F1  RBBP4  B-myb  FOXM1  PLK1  PLK4 |  |  |  |

**siRNAs**

| **Name** | **Sequences** |
| --- | --- |
|  |  |
| Negative control siRNA | Sense: 5'-UUCUCCGAACGUGUCACGUTT-3'  Antisense: 5'-ACGUGACACGUUCGGAGAATT-3' |
| DEPDC1 siRNA | Sense: 5'-GGAAGAUGUUGAAGAAGUUTT-3'  Antisense: 5'-AACUUCUUCAACAUCUUCCTT-3' |

**shRNAs**

| **Name** | **Sequences** |
| --- | --- |
| Negative control shRNA | Sense: *AGCTT*GTTCTCCGAACGTGTCACGTTT  CAAGAGAACGTGACACGTTCGGAGAATTTTTT*A*  Antisense: *GATCT*AAAAAAACGTGACACGTTC  GGAGAATCTCTTGAATTCTCCGAACGTGTCACGTC*A* |
| DEPDC1shRNA | Sense: *AGCTT*GGGAAGATGTTGAAGAAGTTTTC  AAGAGAAACTTCTTCAACATCTTCCTTTTTT*A*  Antisense: *GATCT*AAAAAACTTCTTCAACATCT  TCCTCTCTTGAAACTTCTTCAACATCTTCCC*A* |
