# Supplementary material for: DEPDC1 is required for cell cycle progression and motility in nasopharyngeal carcinoma
Source: Oncotarget. 2017 Jun 29;8(38):63605–19. doi: 10.18632/oncotarget.18868 (PMC5609947; doi:10.18632/oncotarget.18868)
Supplement: Supplementary file 4 [file oncotarget-08-63605-s004.docx]

**Supplementary Table 4: Antibodies used in the present study**

| **Protein**  **name** | **Maneufacture**  **(cat. number)** | **Applications**  **(working dilution)** | | **Website link** |
| --- | --- | --- | --- | --- |
| GAPDH | Xianzhi Bio(AB-P-R 001) | IB(1:5000) | http://www.goodhere.com/showproduct.asp?id=320&classid=34&nid=2 | |
| DEPDC1A | Novus  (26630002) | IF(1:400)  IHC（1:500） | https://www.novusbio.com/products/depdc1a-antibody_26630002 | |
| DEPDC1  E-cadherin  N-cadherin  Vimentin  Twist1  **α**-Tubulin  pHH3  Anti-BrdU  antiMouse seccondary antibody  antiRabbit seccondary antibody  antiMouse seccondary antibody | Thermofisher  (PA5-34864)  Origene  (TA800692)  Origene  (TA503933)  CST  (D21H3 #5741)  Abcam  (ab175430)  Abcam  (ab7291)  CST  (9706)  Sigma  (B2531)  Abgent(ASS1007)  Abgent(ASS1009)  Thermo(A-11031) | WB(1:1000)  WB(1:1000)  WB(1:1000)  WB(1:1000)  WB(1:1000)  IF(1:2000)  IF(1:200)  IF(1:1500)  IB(1:5000)  IB(1:5000)  IF(1:1000) | https://www.thermofisher.com/antibody/product/DEPDC1-Antibody-Polyclonal/PA5-34864  http://www.origene.com.cn/antibody/TA800692.aspx  http://www.origene.com.cn/antibody/TA503933.aspx  https://www.cellsignal.com/products/primary-antibodies/vimentin-d21h3-xp-rabbit-mab/5741?N=4294956287&Ntt=Vimentin&fromPage=plp  http://www.abcam.cn/twist-antibody-10e4e6-ab175430.html  http://www.abcam.cn/alpha-tubulin-antibody-dm1a-loading-control-ab7291.html  https://www.cellsignal.com/products/primary-antibodies/phospho-histone-h3-ser10-6g3-mouse-mab/9706?N=4294956287&Ntt=Phospho+Histone+H3++ser10&fromPage=plp  http://www.sigmaaldrich.com/catalog/product/sigma/b2531?lang=zh&region=CN  http://www.abgent.com/products/ASS1007-Goat-Anti-Mouse-IgGHL-Human-ads-HRP-Secondary-Antibody  http://www.abgent.com/products/ASS1009-Goat-Anti-Rabbit-IgGHL-MouseHuman-ads-HRP-Secondary-Antibody  https://www.thermofisher.com/antibody/product/Goat-anti-Mouse-IgG-H-L-Secondary-Antibody-Polyclonal/A-11031 | |
